# Measuring resting cerebral haemodynamics using MRI arterial spin labelling and transcranial Doppler ultrasound: Comparison in younger and older adults

**DOI:** 10.1002/brb3.2126

**Published:** 2021-05-25

**Authors:** Claire V. Burley, Susan T. Francis, Anna C. Whittaker, Karen J. Mullinger, Samuel J.E. Lucas

**Affiliations:** ^1^ Centre for Human Brain Health University of Birmingham Birmingham UK; ^2^ School of Sport, Exercise and Rehabilitation Sciences University of Birmingham UK; ^3^ Dementia Centre for Research Collaboration School of Psychiatry University of New South Wales Sydney Australia; ^4^ Sir Peter Mansfield Imaging Centre School of Physics and Astronomy University of Nottingham UK; ^5^ Faculty of Health Sciences and Sport University of Stirling Stirling UK; ^6^ School of Psychology University of Birmingham UK

**Keywords:** Aging, cerebral blood flow, cerebral hemodynamics, MRI functional, multimodal imaging, transcranial Doppler sonography

## Abstract

**Introduction:**

Resting cerebral blood flow (CBF) and perfusion measures have been used to determine brain health. Studies showing variation in resting CBF with age and fitness level using different imaging approaches have produced mixed findings. We assess the degree to which resting CBF measures through transcranial Doppler (TCD) and arterial spin labeling (ASL) MRI provide complementary information in older and younger, fit and unfit cohorts.

**Methods:**

Thirty‐five healthy volunteers (20 younger: 24 ± 7y; 15 older: 66 ± 7y) completed two experimental sessions (TCD/MRI). Aging and fitness effects within and between imaging modalities were assessed.

**Results:**

Middle cerebral artery blood velocity (MCAv, TCD) was lower and transit time (MRI) slower in older compared with younger participants (*p* < .05). The younger group had higher gray matter cerebral perfusion (MRI) than the older group, albeit not significantly (*p* = .13). Surprisingly, fitness effects in the younger group (decrease/increase in MCAv/transit time with fitness, respectively) opposed the older group (increase/decrease in MCAv/transit time). Whole cohort transit times correlated with MCAv (r=−0.63; *p* < .05), whereas tissue perfusion did not correlate with TCD measures.

**Conclusion:**

TCD and MRI modalities provide complementary resting CBF measures, with similar effects across the whole cohort and between subgroups (age/fitness) if metrics are comparable (e.g., velocity [TCD] versus transit time [MRI]).

## Graphical abstract

We assess the degree to which resting CBF measures from transcranial Doppler (TCD) and arterial spin labeling (ASL) MRI provide complementary information in older and younger, fit and unfit cohorts. Middle cerebral artery blood velocity (MCAv, TCD) was lower and transit time (MRI) slower in older compared with younger participants, though fitness effects in the younger group opposed those in the older group. Whole cohort transit times correlated with MCAv, whereas tissue perfusion did not correlate with TCD measures.

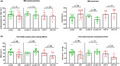

## 1 INTRODUCTION

Resting cerebral blood flow (CBF) and perfusion measures have been used to determine brain health. Perfusion abnormalities are associated with dementia and mild cognitive impairment (Alexopoulos et al., 2012), and lower CBF is associated with accelerated cognitive decline and increased risk of dementia (Wolters et al., 2017). Studies measuring resting CBF have used different methodological approaches to determine this outcome measure, including the choice of imaging modality to collect the data as well as the analysis approach or metric used to determine the resting CBF. Given the physiological and physical differences between approaches (i.e., phenomena being measured such as cerebral hemodynamics and/or volumetric measures), it is important to consider whether the results obtained reveal similar differences between groups irrespective of modality acquisition and analysis approach or metric used. This is vital to enable valid interpretation of outcome measures obtained across different imaging modalities on different patient cohorts.

Two of the most common neuroimaging modalities for measuring resting CBF are transcranial Doppler (TCD) ultrasound and arterial spin labeling (ASL) magnetic resonance imaging (MRI). TCD‐based measures include middle cerebral artery blood velocity (MCAv) and/or cerebrovascular conductance (CVCi) [calculated by dividing MCAv by mean arterial blood pressure (MAP)] and are often used to assess resting CBF (Brown et al., 2010). ASL MRI measures are commonly used to assess cerebral perfusion across all gray matter tissue (global perfusion) or gray matter tissue within specific regions of interest (RoIs), with some ASL sequences using multiple inversion times to estimate blood transit times to the tissue (Alsop et al., 2015).

The MCA is the largest branch of the internal carotid artery (ICA) and supplies the frontal, temporal, and parietal lobes of the brain. This makes it an ideal blood vessel to investigate blood flow to the brain and is often targeted with TCD. Elevated blood pressure (i.e., hypertension) has been linked to cognitive impairment (Elias et al., 2012) and therefore is important to consider when investigating resting CBF. Concurrent recordings of mean arterial blood pressure (MAP) enable researchers to calculate cerebrovascular conductance indices, with this metric providing an additional measure of resting CBF that has the benefit of accounting for changes in CBF driven by blood pressure (Harper, 1966).

ASL MRI measurement of resting CBF can provide quantitative measures of resting cerebral perfusion and blood flow transit times (Alsop et al., 2015). Cerebral perfusion refers to the rate at which blood is delivered to the capillary bed (Liu and Brown 2007) and is commonly expressed in milliliters of blood per 100 grams of tissue per minute (mL·100g^‐1^·min^‐1^). Transit times refer to the time taken for blood to travel to various regions of the brain from the labeling plane (typically at the top of the neck/skull base) where the blood has been “tagged.” One important consideration with ASL measures is that, if not accounted for, cortical atrophy due to healthy aging will affect measures of cerebral perfusion due to an overall reduction in tissue (Chen et al., 2011). If transit times are not accounted for then incorrect estimates of perfusion may be obtained as the images may not be acquired at the correct time relative to the “tag” inversion pulse (Alsop et al., 2015). This is particularly pertinent if two cohorts with different transit times are acquired with the same label delay. Therefore, it is important to acquire data at multiple inversion times to allow the estimation of transit times and related resting perfusion measures so that aging effects on blood perfusion are fully accounted for (Parkes et al., 2004).

Metrics of resting CBF across modalities have generally been shown to decline in healthy aging with these effects offset by greater physical fitness. This has been demonstrated in studies using TCD (Ainslie et al., 2008; Bailey et al., 2013; Barnes et al., 2013; Brown et al., 2010) and ASL MRI imaging methods (Bastos‐Leite et al., 2008; Zimmerman et al., 2014); although conflicting findings have recently been reported from MRI‐based studies showing that lower resting CBF (perfusion measured with ASL) was associated with *higher* cardiorespiratory fitness (Furby et al. 2019; Intzandt et al., 2019). Furthermore, resting CBF has been reported to be impaired in cerebrovascular disease (ASL MRI: Detre et al., 1998) and neurocognitive conditions compared with the healthy population, including stroke (TCD: Markus and Cullinane, 2001), dementia (TCD: den Abeelen et al., 2014, and ASL MRI: Alexopoulos et al., 2012; Alsop & Detre 1996 & Alsop et al., 2000; Wolters et al., 2017; Zhang et al., 2017), and traumatic brain injury (ASL MRI: Kim et al., 2010).

These previous studies have not made these observations in the same participant cohort(s) across modalities, limiting the ability to draw cross‐modal inferences. Since TCD and ASL MRI target different aspects of the vascular tree, TCD is targeting a specific point in a blood vessel (e.g., the central area of the MCA), whereas ASL is measuring perfusion in the tissue consisting of a host of microvessels; it cannot be assumed that measures from the two modalities will directly relate. In addition, with multiple metrics reported from each modality (TCD: MCAv, CVCi; ASL: cerebral perfusion, transit time), it is unclear which will most closely relate across modalities. Therefore, it is important to systematically consider whether these differences between modalities and metrics alter interpretation of the differences in resting CBF measures between groups depending on the imaging modality used to assess resting CBF. As such, the purpose of this study was to examine whether these methodological differences alter the relative outcome measures of resting CBF between groups where differences in resting CBF are expected (i.e., younger/older and fit/unfit).

### 1.1 Study aims and hypotheses

The overall aims of this study were to: (1) investigate differences between age (younger versus older) and fitness (fit versus unfit) groups in resting CBF outcome measures across imaging modalities, and (2) determine whether differences in imaging modality (i.e., TCD versus MRI), associated analysis approaches and metrics influence the resting CBF outcome measure and thus the relationship across modalities.

Based on the previous work (Alsop & Detre 1996 & Alsop et al., 2000; Zhang et al., 2017, 2018) in either modality separately, it was hypothesized that: (1) TCD and MRI assessment of resting CBF would provide a similar pattern between younger versus older, and fit versus unfit participants, where younger participants would have higher resting CBF measures than older participants, and fit participants would have higher resting CBF than unfit participants, and (2) resting CBF measures obtained using the TCD modality would correlate with resting CBF measures obtained using the MRI modality across the whole group.

## 2 MATERIALS AND METHODS

Ethical approval was obtained for all experimental protocols and procedures by the University of Birmingham Ethics Committee and the study conformed to the Declaration of Helsinki (project code: ERN_14‐1423). Participants completed five visits on separate days to either the School of Sport, Exercise and Rehabilitation Sciences (four visits) or the Birmingham University Imaging Centre (one visit) at the University of Birmingham. Prior to participation, a detailed verbal and written explanation of the study was provided, and written informed consent was obtained.

### 2.1 Participants

Thirty‐five healthy volunteers in two age groups participated: 20 younger participants, mean age 24 ± 7 years and 15 older participants, mean age 66 ± 7 years. Participants were excluded if they had any neurological or psychiatric conditions or if any abnormalities were revealed from a 12‐lead electrocardiogram (ECG). Groups were further divided into fit and unfit groups, as determined by performance on a maximum oxygen consumption (V̇O_2_max) fitness test (see below for details). The partitioning of fitness for each age group was as follows: For younger participants, a V̇O_2_max greater than 45 ml·min^‐1^·kg^‐1^ placed them in the fit group; for the older participants, a V̇O_2_max greater than 25 ml·min^‐1^·kg^‐1^ placed them in the fit group. Remaining participants were placed in the associated unfit groups. Partitioning values were based on normative data, which includes the well‐established decline in cardiorespiratory fitness across the lifespan (Heyward, 1998; Riebe et al., 2018).

### 2.2 Experimental visits

#### 2.2.1 Overview

Participants completed five visits in total. The first visit included general health screening, MRI safety screening, fitness questionnaires, and an electrocardiogram (if over 50 years of age). The second visit included the aerobic fitness test on a treadmill or stationary bike. The third visit was a familiarization of the CBF measures using TCD and gas challenges (reported elsewhere; see Burley et al., 2021). The final two visits involved collecting CBF measures, one using TCD and the other using MRI. The order of these final two visits was randomized and counterbalanced between participants using a computer random number generator. For all visits, participants were asked to avoid vigorous exercise and alcohol for 24 hr, caffeine for 12 hr, and heavy meals for 4 hr prior to study participation.

#### 2.2.2 Electrocardiogram and general health screening

Participants underwent a pre‐exercise evaluation for general health screening. Participants > 50 years completed a resting 12‐lead ECG assessment and resting blood pressure measurement, which was reviewed by a cardiologist. Exclusion criteria included family history of heart attack, high resting blood pressure (systolic > 160, diastolic > 90), and ECG abnormalities (S‐T suppression, >3 ectopic beats in a row, referral to GP advised). Participants were not taking any medication and had no history of cardiovascular, cerebrovascular, or respiratory disease.

#### 2.2.3 Aerobic fitness assessment

Aerobic fitness was determined from a maximal oxygen consumption (V̇O_2_max) test. After screening and inclusion into the study, all participants completed a maximal aerobic fitness test to determine V̇O_2_max. Participants could choose to either cycle on an electromagnetically braked cycle ergometer or run on a treadmill. Although these approaches have been shown to yield different VO_2_max values (Loftin et al., 2004), we considered the choice was justified so that older adults who were more likely to be frail or have other movement limitations (particularly those who were less fit) were more likely to be able to complete the test. The respiratory exchange ratio (RER), heart rate, and rate of perceived exertion (RPE) were all monitored throughout to determine a valid fitness test (Riebe et al., 2018).

For the cycling protocol, participants were asked to cycle at a rate of ~ 70 rpm (rotations per minute) or above throughout, while the workload increased in increments of between 20 and 35 Watts (depending on age and fitness) every three minutes. For the running protocol, initial pace began at a speed where heart rate was approximately 65% of their predicted maximum, and then increased by 0.5 to 1.0 km·hour^‐1^ (depending on age and fitness) every two minutes for the first four stages, and then at a 1% incline per minute. For both protocols, the workload continued to increase until the participant reached volitional exhaustion or heart rate reached 100% of the participant's estimated maximum heart rate. Respiratory gases and gas volume were collected for measurement of the rate of VO_2_. V̇O_2_max was then calculated from a 30‐s average around the peak VO_2_ (i.e., highest value) and divided by body weight (kilograms).

#### 2.2.4 Resting cerebral blood flow and perfusion outcome measures

All resting measures were recorded from the period during which participants were breathing room air following at least 20 min of supine rest, at the same time of day. For the TCD session, the supine rest period included locating the right and left MCA and complementary physiological measures (described below). For the MRI visit, the supine period consisted of familiarization with the scanning environment and standard set‐up scans required for planning the experimental scans.

### 2.3 Data acquisition

*TCD session:* Blood velocity in the right and left middle cerebral artery (MCAv) was measured using TCD (Doppler Box, DWL, Compumedics Ltd, Germany), with a 2‐MHz probe placed over each temporal window on the right and left side of the head. Probes were prepared with ultrasound gel and held in place with a headset. Search and identification procedures were done in accordance with established guidelines (Willie et al., 2011). Beat‐by‐beat blood pressure (BP) was measured using photoplethysmography via a finger cuff placed on the middle finger of the left hand (Portapres, Finapres, Medical System BV, Netherlands). A 3‐lead electrocardiogram (ECG) was used to continuously measure heart rhythm and electrical activity. The partial pressure of end‐tidal carbon dioxide (Petco_2_ ) was sampled breath by breath from a mouthpiece that participants breathed through during testing, with a sample line attached to the mouthpiece and an online fast‐responding gas analyzer (ML206, ADInstruments Ltd, Dunedin, New Zealand). The gas analyzer was calibrated before each testing session and end‐tidal values corrected for barometric pressure of the day to allow valid comparison between sessions. These data were recorded via an analogue‐to‐digital converter (Powerlab, ADInstruments) using LabChart software (v7, ADInstruments).

*MRI session:* All MRI data were acquired on a 3‐T Philips Achieva MRI scanner (Philips Medical Systems, Best, The Netherlands). A whole‐body transmit coil and 32 channel head receive coil were used for all data acquisition. To allow quantification of resting perfusion using MRI, images were acquired using a flow‐sensitive alternating inversion recovery (FAIR) pulsed ASL sequence with two‐dimensional echo‐planar imaging (2D‐EPI) readout Kim, 1995). This scan lasted ten minutes. The imaging parameters were echo time (TE): 9 ms; repetition time (TR): 8 s; inversion times (TIs): 0.4, 0.6, 0.8, 1.0, 1.2, 1.4, 1.6, and 1.8 s; voxel size: 3.25 × 3.25 mm in plane; slice thickness: 5 mm; slices: 12; field of view (FOV): 212 x 212 mm; no background suppression or vascular crushing; and sensitivity encoding in parallel imaging (SENSE factor): 2.5. Four volumes of data were acquired for TIs of 0.4 → 1.4 s while 10 volumes of data acquired for TIs of 1.6 → 1.8 s, due to lower signal to noise at longer TIs. A base equilibrium M_0_ scan was acquired with the same parameters but without the inversion pulses required for ASL sequence. Slices were positioned axially from the motor cortex and angled (anterior–posterior) to cover as much of the cortex as possible. A whole head T1‐weighted anatomical image (MPRAGE) with 1 mm^3^ resolution was also acquired to allow definition and segmentation of the gray and white matter so that resting CBF measures in gray matter could be assessed. Cardiac and respiratory cycles were simultaneously recorded using the scanner's physiological monitoring system (VCG and respiratory belt) whose outputs are sampled at 500 Hz. The Petco_2_ was sampled breath by breath from a mouthpiece that participants breathed through during testing, with a sample line attached to the mouthpiece and an online fast‐responding gas analyzer (AEI Technologies, Pittsburgh, PA). The gas analyzer was calibrated before each testing session and end‐tidal values corrected for barometric pressure of the day to allow valid comparison between sessions. Similar to the TCD session, Petco_2_ data were recorded via a Powerlab analogue‐to‐digital converter (ADInstruments) using LabChart software (v7, ADInstruments).

### 2.4 Data analysis

*TCD data:* Mean resting MCAv (cm·s^‐1^) data were extracted from a 60‐s duration sample within the final 5 min of the resting recording. CVCi was calculated by dividing the mean MCAv by the mean MAP during the same 60‐s duration.

Heart rate was calculated from the ECG trace acquired during the TCD acquisition and averaged over a 1‐min duration during baseline. Petco_2_ was obtained from the peak of the expired breath trace recorded within LabChart. These values were compared with the HR and Petco_2_ measures taken from the MRI data acquisition visit.

*MRI data:* Data were averaged over repeats for each TI. Using FSL BET (https://fsl.fmrib.ox.ac.uk/fsl/fslwiki), the brain was extracted from the anatomical image and gray matter mask created for each participant (Smith, 2002). CBF data were coregistered to the participant's extracted brain image. The extracted brain image from the MPRAGE data was then normalized to Montreal Neurological Institute (MNI) standard brain (MNI152_T1_2mm_brain) using FSL FLIRT (Jenkinson and Smith, 2001; Jenkinson et al. 2002; Greve & Fischl 2009). This transformation was then applied to the coregistered resting CBF data and gray matter mask for each participant.

Resting cerebral perfusion (mL·100g^‐1^·min^‐1^) and transit time (seconds) measures were calculated using the FSL Bayesian Inference for Arterial Spin Labelling MRI (BASIL) toolset (Chappell et al., 2009: https://fsl.fmrib.ox.ac.uk/fsl/fslwiki/BASIL). ASL data acquired at multiple TIs were fitted to the kinetic curve model (Lu et al., 2004) so that perfusion estimation errors associated with variable transit times across participant groups could be avoided. The parameters input to the models (based on measured literature values and the ASL sequence used: Wong et al., 1998) were as follows: bolus duration: 1.0 s; bolus arrival time (BAT): 0.8 s; tissue relaxation time (T_1_): 1.3 s; blood relaxation time (T_1_b): 1.65 s (30); timing between slices: 19 ms; label efficiency (alpha): 0.98 (31); T_1CSF_ (reference tissue): 3.3 s (at 3T); T_2CSF_ (reference tissue): 0.3 s; T_2blood_: 0.15 s; and echo time (TE): 9 ms.

Perfusion and transit times were assessed in all gray matter tissue as well as several regions of interest (RoIs) within the gray matter. RoI masks (Figure S1) were defined from the conjunction of the relevant regions from the Harvard atlas (in FSL) and the normalized individual participant's gray matter mask (Figure S1). RoIs used were as follows: cingulate gyrus, frontal lobe, motor lobe, occipital lobe, and parietal lobe, as previously employed (Thomas et al., 2013). Mean cerebral perfusion and transit times across the participant groups were determined for the whole of the imaged gray matter and the different RoIs. In addition, mean cerebral perfusion and transit time group maps were created for all participants and the younger and older groups separately by averaging the participant's individual mean perfusion and transit time maps (Figures S3 and S4).

Heart rate was calculated from the VCG trace acquired during the ASL acquisition and averaged over the whole time period. P_ET_CO_2_ was obtained from the peak of the expired breath trace recorded within the LabChart software. These values were compared with the same measures taken from the TCD data acquisition visit.

### 2.5 Statistical analysis

The researcher was blind to the age and fitness level of the participant data during analysis. The analysis included typical approaches to calculate resting measures of CBF obtained from MRI cerebral perfusion and transit times (Chen et al., 2011; Parkes et al., 2004) and TCD MCAv and CVCi (Brown et al., 2010), and measures of resting MAP and HR. Means were calculated for younger and older groups and fit and unfit subgroups (younger fit and younger unfit; older fit and older unfit). Separate one‐way ANOVAs were used to examine effects of aging and aerobic fitness on resting CBF measures. When examining age effects, age group (younger versus older) was the factors and dependent variables were the resting CBF measures. Post hoc correlational analysis (Spearman's *r*) was then performed where outcome measures were also correlated against age for all participants together, and against fitness separately for the younger and older groups. Resting CBF measures using different imaging modalities were compared using Spearman's *r* correlations. A *p* value less than 0.05 was considered statistically significant. To correct for multiple comparisons when correlating TCD and MRI measures, we adjusted the *p* value by dividing by the number of comparisons (McDonald, 2009), not including the supplementary analysis of the individual RoIs (adjusted *p* value = 0.05/4 = 0.0125).

## 3 RESULTS

Thirty‐five participants completed the resting TCD measures. Thirty‐three participants completed all the resting TCD and MRI CBF measures. One participant chose not to complete the MRI visit, and one MRI dataset was lost due to technical issues. Mean participant characteristics from the first TCD visit are reported in Table 1, including: age, fitness (V̇O_2_max), heart rate (HR), and MAP.

**TABLE 1:** Characteristics of participants who completed the resting CBF measures using TCD and MRI brain imaging modalities

Group (*n*)	Younger (20)	Older (15)	*p* value	Younger fit (8)	Younger unfit (12)	*p* value	Older fit (9)	Older unfit (6)	*p* value
Age (years)	24.5 (6.9)	**66.5** ** (6.7)	<0.001	27.6 (8.1)	22.4 (5.3)	0.097^t^	64.2 (6.8)	69.8 (5.4)	0.114
V̇O_2_max (mL·min^−1^·kg^−1^)	43.8 (10.7)	**33.4** * (15.9)	0.026	54.5 (5.0)	**36.0** ** (5.5)	<0.001	42.6 (13.9)	**19.7** ** (3.3)	0.001
Heart Rate (b·min^−1^)	64 (8)	57 (8)	0.124	55 (7)	**70** * (15)	0.018	55 (7)	61 (8)	0.181
MAP (mm Hg)	80 (11)	83 (11)	0.445	77 (12)	82 (9)	0.297	80 (9)	89 (12)	0.120
Sex (male: female)	12:8	10:5		7:1	5:7		8:1	2:4	

Note

Values represent mean (± standard deviation) obtained from ANOVAs. Age groups were defined as younger (18–40 years) and older (50–80 years), while the criterion for being fit was defined as ≥ 45 ml·min^‐1^·kg^‐1^ and ≥ 25 ml·min^‐1^·kg^‐1^ for the younger and older groups, respectively. Heart rate values are from the TCD visit.

Abbreviations: MAP, mean arterial blood pressure; V̇O_2_max, maximum oxygen consumption.

Significant age/fitness effects (different from younger group/ different from fit group)

^t^ Shows a trend toward significance: 0.05 < *p* ≤ 0.10

* *p* ≤ .05

** *p* ≤ .01

Resting heart rate and P_ET_CO_2_ values can influence CBF measures. Comparison of heart rate and P_ET_CO_2_ between TCD and MRI visits was made to ensure there were no systematic differences and values were similar. Paired *t* tests showed there were no significant differences in resting P_ET_CO_2_ (41.6 mm Hg ± 3.4 versus. 41.0 ± 3.9 mm Hg for TCD and MRI, respectively; *p* = .528) or resting heart rate (61 ± 13 versus. 60 ± 12 beats·min^‐1^ for TCD and MRI, respectively; *p* = .669) between the TCD and MRI visits.

### 3.1 Age, fitness and resting cerebral blood flow (CBF) outcome measures

**FIGURE 1:**
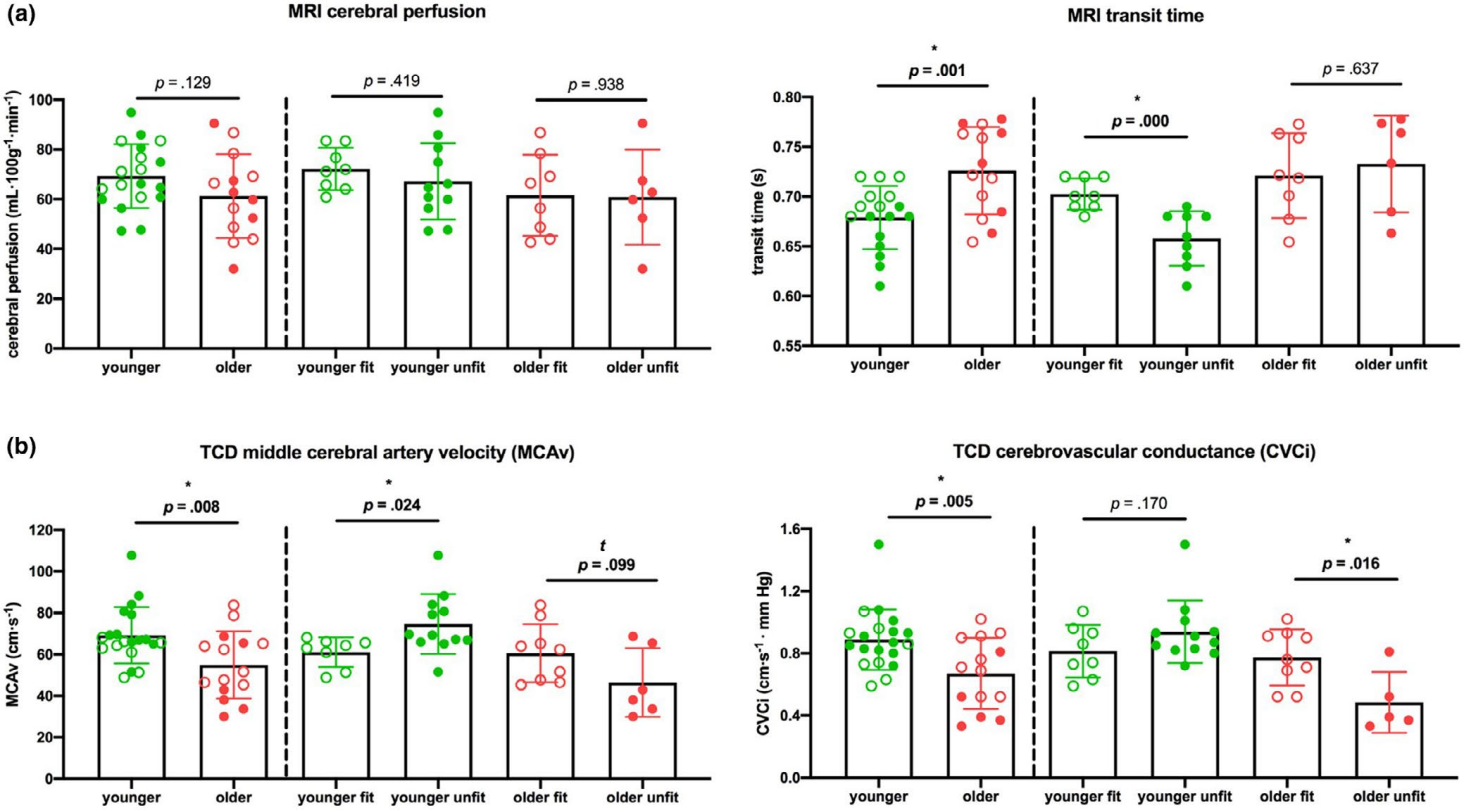
Graphs show mean and individual participant points for resting CBF measures obtained from (a) MRI data (gray matter cerebral perfusion and transit times; see Figure S1.B for gray matter mask) and (b)TCD data (MCAv and CVCi), summarizing results from Table 2. Significance tested with one‐way ANOVAs: *Represents significant effect: *p* ≤.05, ^t^ Shows a trend toward significance: 0.05 < *p* ≤0.10. Error bars show standard deviation. Abbreviations: CVCi, cerebrovascular conductance; MCAv, middle cerebral artery blood velocity; MRI, Magnetic Resonance Imaging; TCD, transcranial Doppler

**FIGURE 2:**
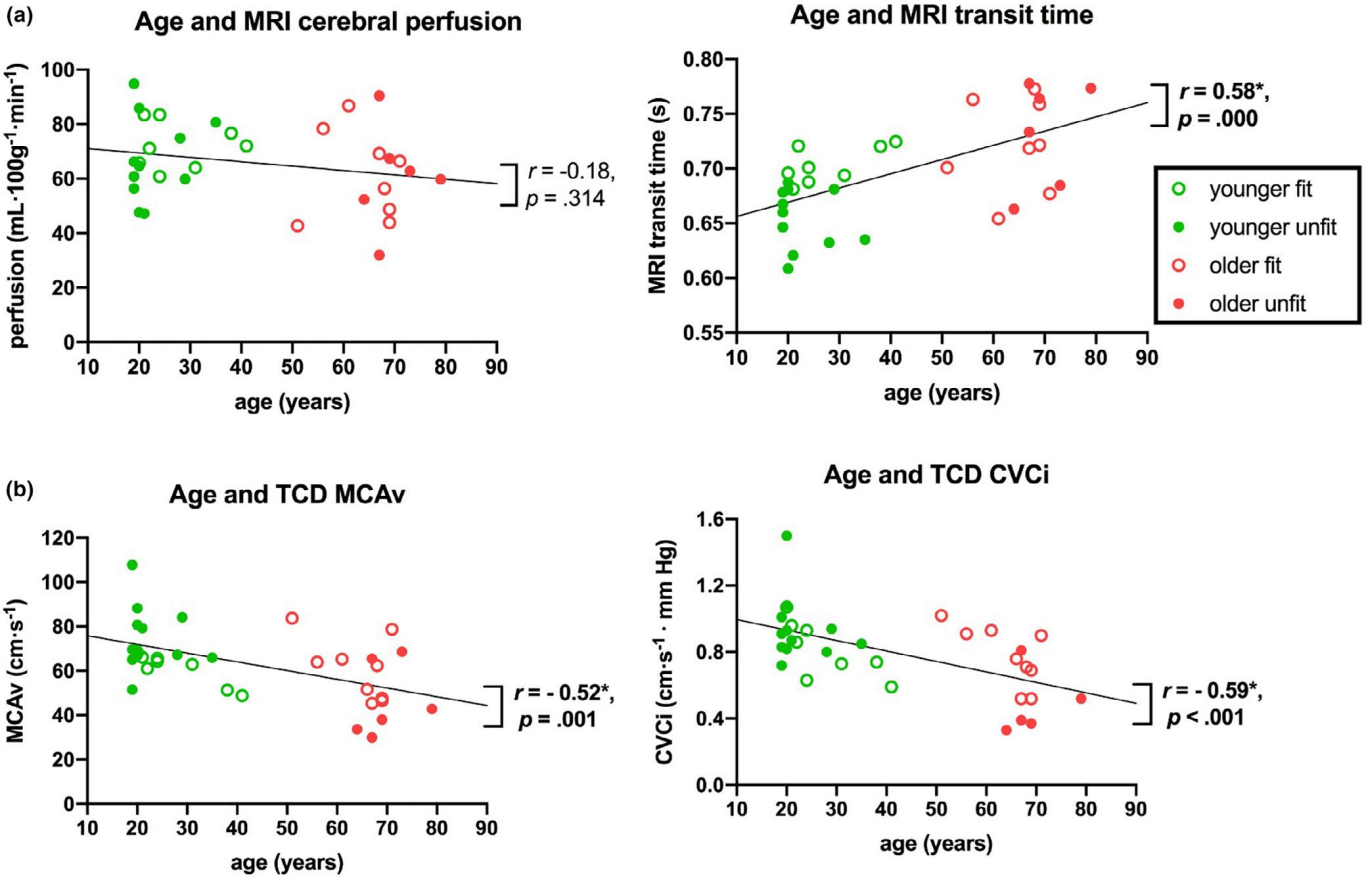
Correlation between age and different resting CBF measures. (a)MRI gray matter (GM) cerebral perfusion and GM transit time and (b)TCD MCAv and CVCi. Black lines denote lines of best fit with Spearman's r and associated *p* values. *Represents significant effect (*p* ≤ .05). Abbreviations: CVCi, cerebrovascular conductance; GM, gray matter; MRI, magnetic resonance imaging; MCAv, middle cerebral artery blood velocity; TCD, transcranial Doppler

**FIGURE 3:**
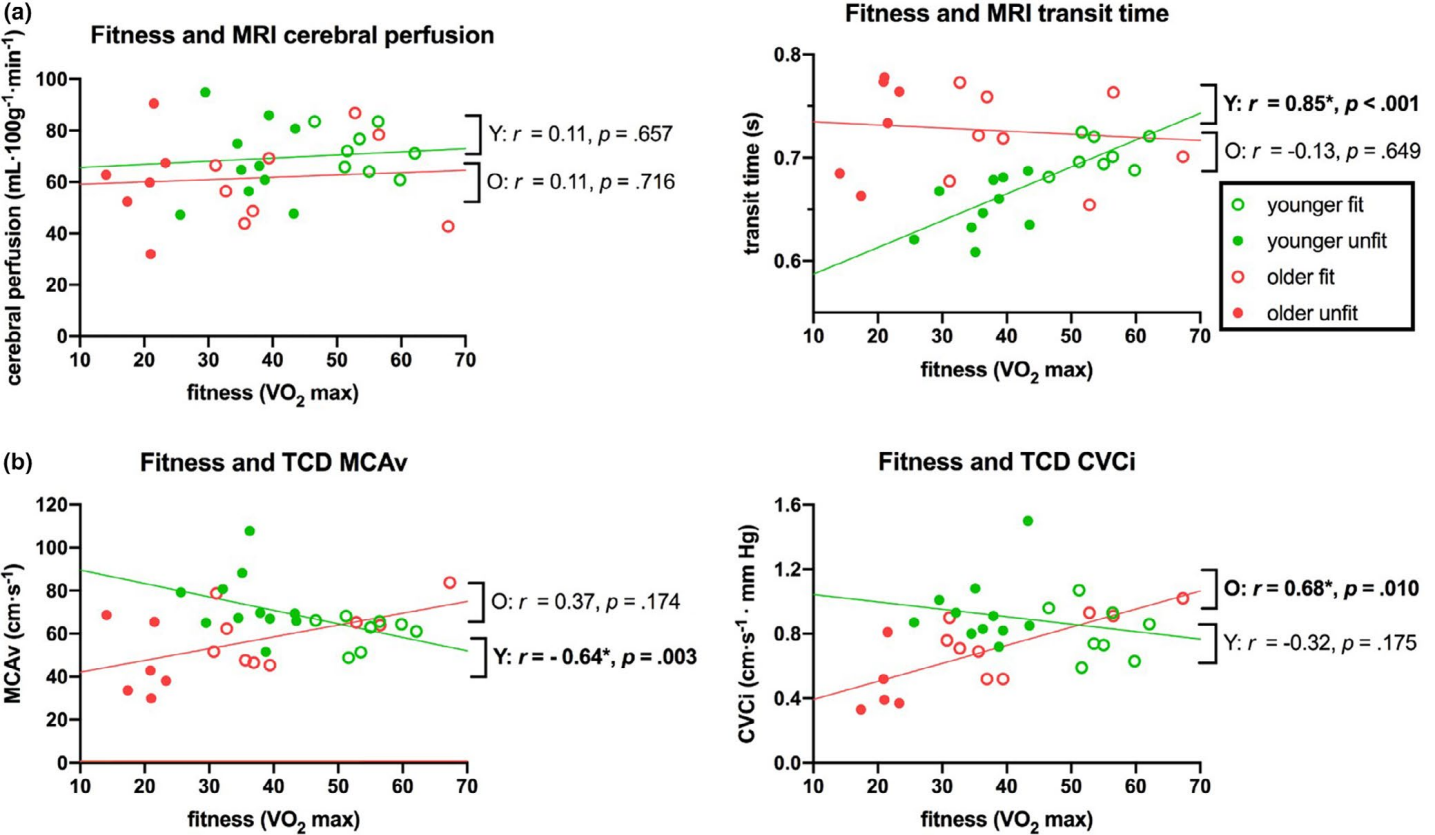
Correlation between fitness (V̇O_2_max) and resting CBF measures, separately for younger (green) and older (red) groups. (a)MRI GM cerebral perfusion and GM transit time, and (b)TCD MCAv and CVCi. Lines of best fit are shown for younger and older groups separately with Spearman's r and significance values. *Represents significant effect (*p* ≤ .05). Abbreviations: CVCi, cerebrovascular conductance; GM, gray matter; MRI, magnetic resonance imaging; MCAv, middle cerebral artery blood velocity; TCD, transcranial Doppler; V̇O_2_max, maximum rate of oxygen consumption

Between‐group outcome measures are shown in Table 2 and Figure 1. Correlations of CBF measures with age and fitness are shown in Figures 2 and 3, respectively.

**TABLE 2:** Mean ± standard deviation for resting CBF measures obtained from MRI data (gray matter cerebral perfusion and transit times) and TCD data (MCAv and CVCi). Measures are shown for all participants followed by groups separated into younger and older; younger fit and unfit; and older fit and unfit

	All participants *n* = 33	By age groups		By age and fitness groups			
		Younger *n*= 1	Older *n*= 16	Younger fit *n*= 9	Younger unfit *n* = 10	Older fit *n* = 9	Older unfit *n* = 7
MRI: GM cerebral perfusion (mL·100g^−1^·min^−1^)	65.9 ± 15.0	69.3 ± 12.9	61.2 ± 16.8	72.2 ± 8.55	67.2 ± 15.3	61.6 ± 16.3	60.8 ± 19.1
MRI: GM transit time (s)	0.70 ± 0.46	0.67 ± 0.34	**0.73 ± 0.44** **	0.70 ± 0.02	**0.65 ± 0.27** **	0.72 ± 0.04	0.73 ± 0.05
TCD: MCA velocity (cm·s^−1^)	63.1 ± 16.2	69.2 ± 13.6	**54.9 ± 16.2** **	61.1 ± 7.14	**74.7 ± 14.4** *	60.6 ± 14.1	**46.4 ± 16.6** ^t^
TCD: CVCi (cm·s^−1^ · mm Hg^−1^)	0.80 ± 0.23	0.89 ± 0.19	**0.67 ± 0.23** **	0.81 ± 0.17	0.94 ± 0.20	0.77 ± 0.18	**0.48 ± 0.19** *

Note

Values represent mean ± standard deviation. Age groups were defined as younger (18–40 years) and older (50–80 years). Significant age/fitness effects shown from ANOVA (different from younger group/ fit group)

^t^ Shows a trend toward significance: 0.05 < *p* ≤ 0.10.

* *p* ≤ .05

** *p* ≤ .01

*MAP and HR data:* No significant differences were observed between younger and older groups for MAP (*p* = .445) or HR (*p* = .124) (Table 1). In line with existing literature (Brown et al., 2010), MAP and HR were on average lower in the fit groups compared to their unfit counterparts for both the older and younger groups, though differences only reached statistical significance for HR in the younger group (see Table 1).

*TCD data:* Significant group differences were observed between younger and older participants for measures of MCAv (*p* = .008) and CVCi (*p* = .005), with the mean of both measures ~ 30% higher in the younger group than the older group (Figure 1). As expected, MCAv and CVCi were higher (31% and 60%, respectively) in the older fit group compared to the unfit group, though was only significant for the CVCi measure (*p* = .016; MCAv: *p* = .099). In contrast, the younger fit group had on average lower MCAv and CVCi (23% and 7%, respectively) compared to their unfit counterparts, though was only significant for the MCAv measure (MCAv: *p* = .024 and CVCi: *p* = .170; Figure 1).

Consistent with these grouped data, further post hoc correlational analysis showed that with increasing age there was a decrease in both MCAv (at a rate of 3.9 cm·s^‐1^ every 10 years) and CVCi (at a rate of 0.06 cm·s^‐1^ · mm Hg every 10 years) (Figure 2b). In the younger group, fitness negatively correlated with MCAv (*r* = −0.64; *p* = .003), while no significant correlation was observed for CVCi (Figure 3b). In contrast, for the older group fitness positively correlated with CVCi (*r* = 0.68; *p* < .010), and no significant correlation was observed for MCAv (Figure 3b).

*MRI data:* In general agreement with the TCD measures, ASL data showed significant group differences between younger and older participants for measures of gray matter transit time, where transit times were on average 7% faster in the younger group (*p* = .001). Gray matter cerebral perfusion was 13% higher in the younger group than the older group, though this difference did not reach statistical significance (*p* = .129). No differences were observed between the older fit and unfit groups for measures of transit times or gray matter cerebral perfusion (Figure 1a). The younger fit group had significantly slower (8%) gray matter transit time compared to their unfit counterparts (*p* < .001; Figure 1a), in agreement with TCD‐based MCAv measures.

Correlational analysis showed that gray matter transit times significantly increased with age (*r* = 0.58, *p* < .001; Figure 2a). In addition, analysis of different RoIs demonstrated that this increase in transit time was widespread, reaching significance for both the ANOVA and correlation analysis in the frontal, motor, and parietal lobes (Table S1 and Figure S2). Age negatively correlated with gray matter cerebral perfusion, though, as with the group analysis, this correlation did not reach statistical significance (*r* = −0.18; *p* = .314; Figure 2a). In the younger group, fitness significantly correlated with gray matter transit time (*r* = 0.85; *p* < .001), where increased fitness was associated with longer transit times (Figure 3a). In the older group, no fitness effects were observed for transit time measures. Perfusion measures showed no fitness effects in the younger or older group (Figure 3a).

### 3.2 Correlations between different approaches of measuring resting cerebral blood flow (CBF)

**FIGURE 4:**
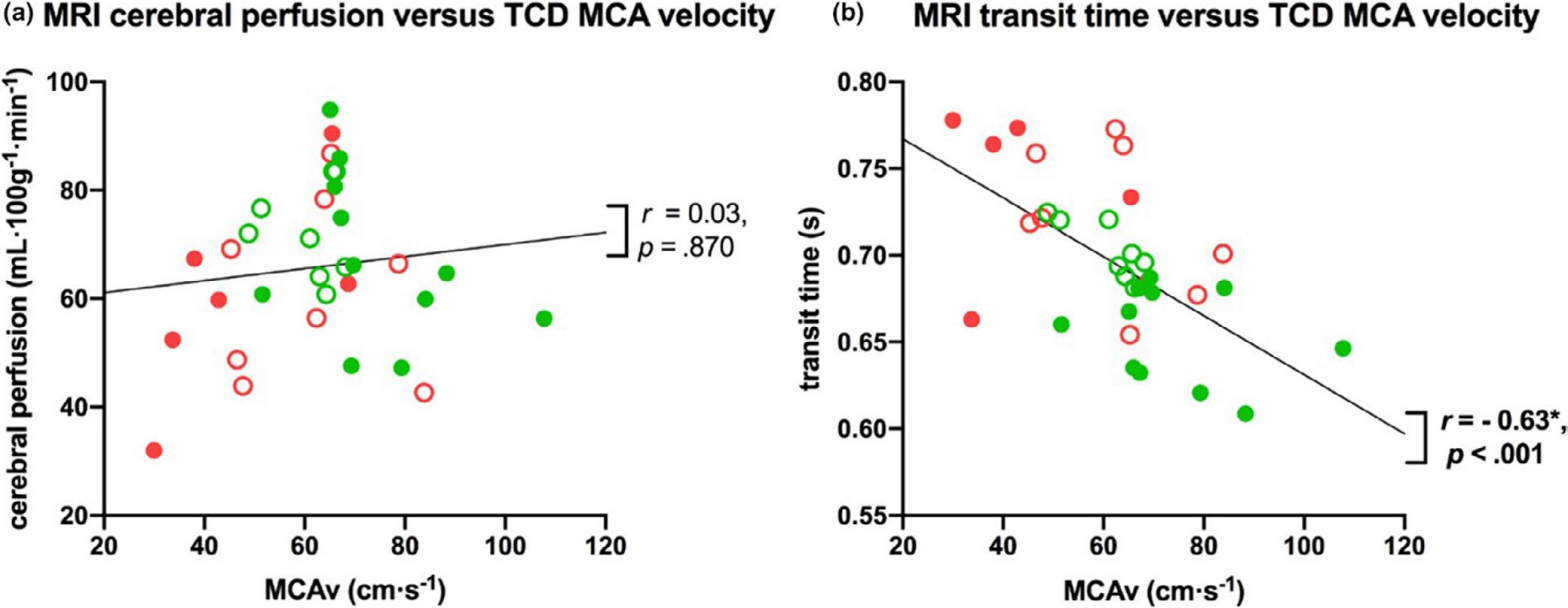
Correlation between resting CBF measures for (a)MRI gray matter perfusion and TCD MCA velocity, and (b)MRI gray matter transit time and TCD MCA velocity. Lines of best fit are shown with Spearman's r and significance values. Abbreviations: MRI, magnetic resonance imaging; MCAv; middle cerebral artery blood velocity; TCD, transcranial Doppler

*ASL transit times and TCD:* ASL‐MRI transit time measures of CBF for all gray matter negatively correlated with all TCD resting CBF measures (MCAv: *p* < .001 and CVCi: *p* = .01) (Figure 4b). In addition, resting MCAv negatively correlated with transit times in all RoIs (all *p* < .05), while transit times in most RoIs also negatively correlated CVCi (Table S1 and Figures S2 and S4), indicating this relationship was not region specific.

*ASL cerebral perfusion and TCD:* MRI perfusion measures for all gray matter (and RoIs, Table S1 and Figure S2 and S3) showed no correlation with TCD‐based CBF resting measures assessed using MCAv and CVCi (Figure 4a).

## 4 DISCUSSION AND CONCLUSIONS

The overall aims of this study were to (1) investigate differences between age (younger versus older) and fitness (fit versus unfit) groups in resting CBF outcome measures across imaging modalities, and (2) determine whether differences in imaging modality (i.e., TCD versus ASL MRI), associated analysis approaches and metrics influence the resting CBF outcome measure and thus the relationship across modalities. This was done by examining group differences in mean outcome measures in younger and older, fit and unfit individuals, with additional post hoc correlational analyses of outcome measures with age and fitness. Further, this study used correlational analyses to investigate whether there were associations between resting CBF measures obtained using different neuroimaging modalities (i.e., ASL MRI and TCD) and measures (i.e., MRI: cerebral perfusion and transit times, and TCD: resting MCAv and CVCi) and whether different approaches would influence the resting CBF outcome measure and its interpretation.

### 4.1 Resting CBF and age

Outcome measures from the TCD modality revealed that resting MCAv and CVCi were significantly lower in the older group compared to the younger group. Outcome measures from the MRI modality revealed that resting CBF perfusion was lower in the older group compared with the younger group in the whole gray matter (and all RoIs), though group differences were only significant in the occipital and parietal lobes. Transit times were longer in the older group compared with the younger group in the whole gray matter and all RoIs (Figure S2 and Table S1; significant for whole gray matter and frontal, motor, and parietal lobes). These findings agree with several previous reports using either TCD (Ainslie et al., 2008; Bailey et al., 2013; Brown et al., 2010; Lucas et al., 2012) or MRI (Liu et al., 2012) and, as hypothesized, show a good agreement across modalities within this same cohort.

The correlational analysis across the entire cohort revealed that as age increased both MCAv and CVCi metrics decreased. Specifically, MCAv decreased at a rate of 3.9 cm·s^‐1^ every 10 years, while CVCi decreased at a rate of 0.06 cm·s^‐1^·mm Hg^‐1^ every 10 years (Figure 2b). This is in line (though not as high) with previous findings (Ainslie et al., 2008), reporting MCAv decreased at a rate of 0.76 cm·s^‐1^ every year (i.e., 7.6 cm·s^‐1^ every 10 years). The difference in the magnitude of change may be related to the much larger sample size used in their study, which had 154 trained and 153 untrained male only participants. In addition, both males and females were included in the current study, which may provide another explanation for lower rate of change across the age range that was compared here given the higher MCAv values observed in females (Marinoni et al., 1998).

Although an age effect on the CBF perfusion measure was not observed in all gray matter, significant group differences in CBF perfusion were observed between younger and older participants in the occipital lobe (37% higher in the younger group; Figure S3 and Table S1). Previous studies have reported age effects of resting perfusion globally (Parkes et al., 2004; Zimmerman et al., 2014) and locally (Chaddock‐Heyman et al., 2016; Thomas et al., 2013). The much subtler effect we observed here may be due to differences in the ASL sequence used, with many previous studies using a fixed postlabel delay (Chaddock‐Heyman et al., 2016; Parkes et al., 2004; Thomas et al., 2013) that does not account for different transit times, which we observe here (Figure 2a), and will make the change in perfusion with age appear greater (Alsop et al., 2015). It is worth noting that the findings of the regional difference could be explained by the occipital region having generally higher perfusion due to vascular structure (Figure S3), which might mean we have more sensitivity to detect differences in these regions due to a higher signal‐to‐noise ratio than other brain regions (Zhou et al., 2015). Therefore, given the same pattern was observed in all brain regions, it may be that this is a global phenomenon that is most pronounced in regions exhibiting the highest perfusion. However, further work is needed to clarify this.

### 4.2 Resting CBF and fitness

Surprisingly, opposing fitness effects were observed between the younger and older groups in resting CBF measures. The older fit group had higher resting CVCi and MCAv, as well as a slightly shorter transit time (Figures 1 and 3). Findings for the older group are in line with the majority of existing literature, which has reported higher resting CBF (either perfusion or MCAv) with greater fitness (Brown et al., 2010; Bailey et al., 2013; Barnes et al., 2013; Zimmerman et al., 2014; Chaddock‐Heyman). We see a clear effect in agreement with these previous reports in our TCD measures (Figures 1 and 3b) with a very weak, nonsignificant, effect in the MRI ASL (Figures 1 and 3a) measures. This weak effect may be due to the screening process that we were required to use (all older participants had to undergo a pre‐exercise evaluation—see methods) meaning even our unfit group was relatively fit. Alternatively, our use of a multi‐inversion time MRI ASL sequence will have ensured a more accurate estimation of perfusion when transit time varies across the group (Alsop et al., 2015) as seen in our data, and therefore, the perfusion effect we observe in our older group may be reduced due to this compared with studies where a single TI has been used (Chaddock‐Heyman et al., 2016).

In contrast, the younger cohort showed significantly higher MCAv in the unfit compared to fit group, which was supported by the differences in CBF transit times (Figures 1 and 3). This finding diverges from the majority of previous work relating resting CBF and fitness over a range of ages (Brown et al., 2010; Ainslie et al., 2008; Bailey et al. Barnes et al. 2013; Zimmerman et al., 2014; Chaddock‐Heyman et al., 2016). However, recent work by others has also observed conflicting findings. For example, Furby and colleagues (2019) found lower resting CBF (perfusion measured with ASL) was associated with *higher* cardiorespiratory fitness in a younger cohort (Furby et al. 2019). While our perfusion measure was not significantly different between our fit and unfit groups, the significant difference in transit times that we observed (which was not reported by Furby *et al*) combined with a slightly different analysis approach may account for this slight difference in results.

Given the relatively large body of work showing greater aerobic fitness is associated with higher resting CBF, further interrogation of our cohort and data were required. One potential explanation for our finding is due to the unbalanced numbers of males and females in our groups. Specifically, there were more males in the fit group, whereas there were more females in the unfit group. Given that previous studies have reported higher resting CBF measures in females compared with males (Marinoni et al., 1998; Parkes et al., 2004), this may explain our findings. Further, the average age of the young fit group was higher than the young unfit group, which may have introduced an age‐related effect over‐and‐above the potential fitness effect.

To try and interrogate these issues, further analysis was performed, specifically looking at the TCD‐based resting MCAv and MRI‐based transit time data where participants were pair matched for age and sex (see Supplementary Results, section SI1). Despite this procedure, the same pattern was observed between fitness groups when considering transit times, although no significant differences were found in the TCD data in these subgroups. This result needs to be considered with caution given the very low number of participants (*n* = 8), and further investigation is required to test for reproducibility of these observations in a larger cohort where participants are closely pair matched for age and sex between fitness groups. However, Furby and colleagues (Furby et al. 2019) only used males in their study and reported results in agreement with our study, indicating this may not be a simple sex or age effect across the group.

An additional consideration in our data and that of Furby and colleagues (Furby et al. 2019) is the relatively small sample sizes used (here we had 10 participants per subgroup while Furby and colleagues reported data from 11 participants in total). Given the sizes of these cohorts, individual variability may be causing these somewhat unexpected findings and could potentially be controlled for in a future study. Nevertheless, as recently suggested by Intzandt and colleagues (2019), the aging and fitness effects on cerebrovascular health are likely to be a complex integration of regulatory factors such as changes in chemo‐sensitivity and autoregulation in addition to changes in arterial stiffness (Intzandt et al., 2019), which seem likely to impact on resting CBF measures and functional stimulus‐response effects (e.g., cerebrovascular reactivity). Therefore, collecting other physiological data (e.g., continuous blood pressure and respiratory gases) alongside CBF measures, for any imaging modality, is needed to assess the full hemodynamic profile and to attempt to disentangle the relative contributions to the differing fitness‐CBF relationships between younger and older participants observed here; but this was beyond the scope of the present study.

### 4.3 TCD compared with ASL MRI resting CBF measures

Significant correlations were observed between resting CBF measures calculated using ASL MRI and TCD approaches when both modalities used a measure related to the velocity of blood as an index of resting CBF [i.e., velocity (cm·s^‐1^) with TCD versus. transit times (s) with ASL]. Indeed, the two imaging approaches did differentiate the younger from the older and the fit from the unfit groups similarly, including the unexpected finding of increased resting CBF (i.e., MCAv and transit times for TCD and MRI, respectively) in the younger unfit group compared to the fit group (see Figure 1). However, there were no associations between ASL MRI measures of cerebral perfusion and TCD measures of CVCi or MCAv. This finding is likely to be due to the fact no significant differences in perfusion with age or fitness were seen over the whole of gray matter. Given that MRI transit time and TCD MCAv are physiologically similar metrics of flow (both effectively measure the velocity of blood traveling through the vasculature), whereas perfusion is measuring the rate at which blood is delivered to the capillary bed (i.e., tissue), it makes sense that these measures may not necessarily strongly correlate (Liu et al., 2012). Importantly, these findings show that the modality and metric used to determine the resting CBF measure can affect interpretation of the measure and its association with age or fitness. It is important to consider this when comparing between studies in future and realize that discrepancy between modalities may reflect differences in the physiology being measured rather than a particular method is more accurate. We suggest that our data indicate that blood velocity to the brain (MRI transit time) and through the major arteries (TCD MCAv) declines with age, but the supply of blood to the tissue (i.e., MRI perfusion) is not affected by age once blood velocity differences are accounted for through a multi‐TI ASL sequence. This indicates the brain compensates for slower blood velocity, perhaps through vessel diameter changes with age, to ensure perfusion is largely maintained.

Phase‐contrast angiography (PCA) MRI provides a metric of blood flow more closely related to that measured with Doppler, both measure blood velocity or flow in a given blood vessel (Oktar et al., 2006; Khan et al. 2017). Despite this, the relationship between PCA MRI and Doppler measures for a given blood vessel has not been reported to be vastly better than the correlation of transit time and MCAv, which we report here (Figure 4b). Indeed, in recent work, the correlation of PCA MRI and Duplex Doppler (linear array transducer allows measurement of both blood velocity and vessel diameter) measures of blood flow in the internal carotid artery was not significant, but similar measures in the vertebral artery were significant (Khan et al. 2017). This suggests that a comparison between the ASL MRI metrics and Doppler measures may be equally valid to comparing PCA MRI and Doppler measures. PCA is not typically the MRI technique of choice for investigating resting CBF, as ASL MRI is normally used to provide regionally specific information regarding resting CBF over the whole cortex rather than a single vessel. Thus, the comparison between ASL MRI and TCD measures in the current study allows a clearer interpretation across previous research studies examining resting CBF between cohorts (Ainslie et al., 2008; Bastos‐Leite et al., 2008; Brown et al., 2010; Zimmerman et al., 2014).

There are several methodological limitations of MRI and TCD. Although a thorough review is beyond the scope of this study, in summary, MRI is more expensive than TCD, can be uncomfortable and affect natural breathing patterns, is not accessible for all (e.g. metal implants and pacemakers exclusion criteria for scanning), and requires participants to lie very still during scanning. In contrast, TCD measures are criticized for lacking information about vessel diameter, are operator dependent and limited to conduit vessel assessments of blood velocity as indices of global flow to the downstream tissue beds.

### 4.4 Study limitations

As mentioned previously, this study recruited more males than females, particularly in the fit groups and this may have affected our findings. The scientific literature reports findings on the effects of sex on resting CBF; specifically, higher resting CBF has been observed in females compared to males in both TCD (Marinoni et al., 1998; Purkayastha and Sorond 2012) and MRI (Parkes et al., 2004) studies. One confounding factor within our study was that the fit younger group was on average older than the unfit group (28 versus. 22 years), meaning that the fitness effects within this group may be less likely to be detected due to the natural decline in resting CBF that occurs during aging (i.e., estimated to be between 4 cm⋅s^‐1^ (data presented) and 8 cm·s^‐1^ (Ainslie et al., 2008; Bailey et al., 2013) every 10 years). However, since the same participants underwent imaging with both modalities (TCD and MRI), then all comparisons across modalities are unaffected by any sex or age imbalances between groups. Further, the sample size although bigger than previous studies was still small given the number of comparisons undertaken and the potential for false positives. We have chosen not to undertake Bonferroni correction of our correlations of resting CBF effects with age and fitness as these were post hoc analyses to further interrogate the group effects seen in our initial ANOVA analysis. We present all results for the reader to draw their own inferences. Other limitations include the partitioning of fitness values being estimated from normative data and the observation that cycling and running can yield different VO_2_max values in the same individuals (Millet et al., 2009). However, VO_2_max data are used for guidance to determine group allocation and are not a primary outcome measure in this study. Further, the differences in the measured VO_2_max between our fitness groups were large (see Table 1), and thus, it seems unlikely that the exercise modality used would have affected the fitness group allocation. Providing participants with a choice of exercise modality meant that more older people could complete the study. Further, this would not have affected the results of the TCD versus MRI comparisons because all participants completed the same measures. We suggest that these results should be interpreted as preliminary and indicative; however, the fact that TCD MCAv and MRI transit times generally show the same patterns across the groups (age and fitness) and correlate with one another (Figure 4) despite them being entirely separate measures indicates the effects observed are likely to be real. Future research in this area would benefit from pair‐matching of participants for both sex and age in order to differentiate the source of the fitness effect on resting CBF, as discussed earlier.

## 5 CONCLUSION

In conclusion, TCD and MRI imaging modalities provide complementary resting CBF measures when assessing similar metrics of flow (e.g., TCD velocity and MRI transit times), where similar differences across the whole cohort and within subgroups were observed across modalities. This strongly indicates that findings between studies using different modalities to assess resting CBF can be compared when healthy participants are being investigated and the metrics of CBF measured are compatible (e.g., blood velocity versus blood transit time). However, measures of MRI cerebral perfusion and associations with transit times and velocity (MCAv and CVCi) were less clear, likely due to the difference in metric being used (rate of blood flow through the vessel versus tissue perfusion in the capillary bed); thus, further research is needed investigating differences within and between modalities and associations with different populations (i.e., younger, older, and disease). The findings in this study cannot be generalized to other imaging modalities for resting CBF or to disease populations; however, they do highlight the need to consider differences in metrics used to assess brain vascular health. Therefore, further investigation is warranted to determine whether TCD and MRI resting CBF measures complement other resting CBF measures obtained using different imaging modalities (e.g., near‐infrared spectroscopy) or data acquisition methods (e.g., PCA MRI), and to determine whether TCD and ASL MRI resting CBF measures remain complementary in specific disease states.

## CONFLICT OF INTEREST

The authors declared no potential conflicts of interest with respect to the research, authorship, and/or publication of this article.

## AUTHOR CONTRIBUTION

CVB, STF, KJM, and SJEL contributed to the conception or design of the work. CVB, STF, ACW, KJM, and SJEL contributed to the acquisition, analysis, or interpretation of data for the work. CVB, STF, ACW, KJM, and SJEL contributed to drafting of the work or revising it critically for important intellectual content. All authors approved the final version of the manuscript. All authors agree to be accountable for all aspects of the work in ensuring that questions related to the accuracy or integrity of any part of the work are appropriately investigated and resolved. All persons designated as authors qualify for authorship, and all those who qualify for authorship are listed.

## 6 ETHICAL APPROVAL

Ethical approval was obtained for all experimental protocols and procedures by the University of Birmingham Ethics Committee and the study conformed to the Declaration of Helsinki (project code: ERN_14‐1423).

## PATIENT CONSENT STATEMENT

Prior to participation, a detailed verbal and written explanation of the study was provided, and written informed consent was obtained.

### PEER REVIEW

The peer review history for this article is available at https://publons.com/publon/10.1002/brb3.2126.

## Supporting information

Supinfo

## Data Availability

The data that support the findings of this study are available from the corresponding author upon reasonable request.
