# Supplementary material for: Measuring resting cerebral haemodynamics using MRI arterial spin labelling and transcranial Doppler ultrasound: Comparison in younger and older adults
Source: Brain Behav. 2021 May 25;11(7):e02126. doi: 10.1002/brb3.2126 (PMC8323033; doi:10.1002/brb3.2126)
Supplement: Supplementary file 1 — Supinfo [file BRB3-11-e02126-s001.docx]

# **Supplementary Information - Methods**


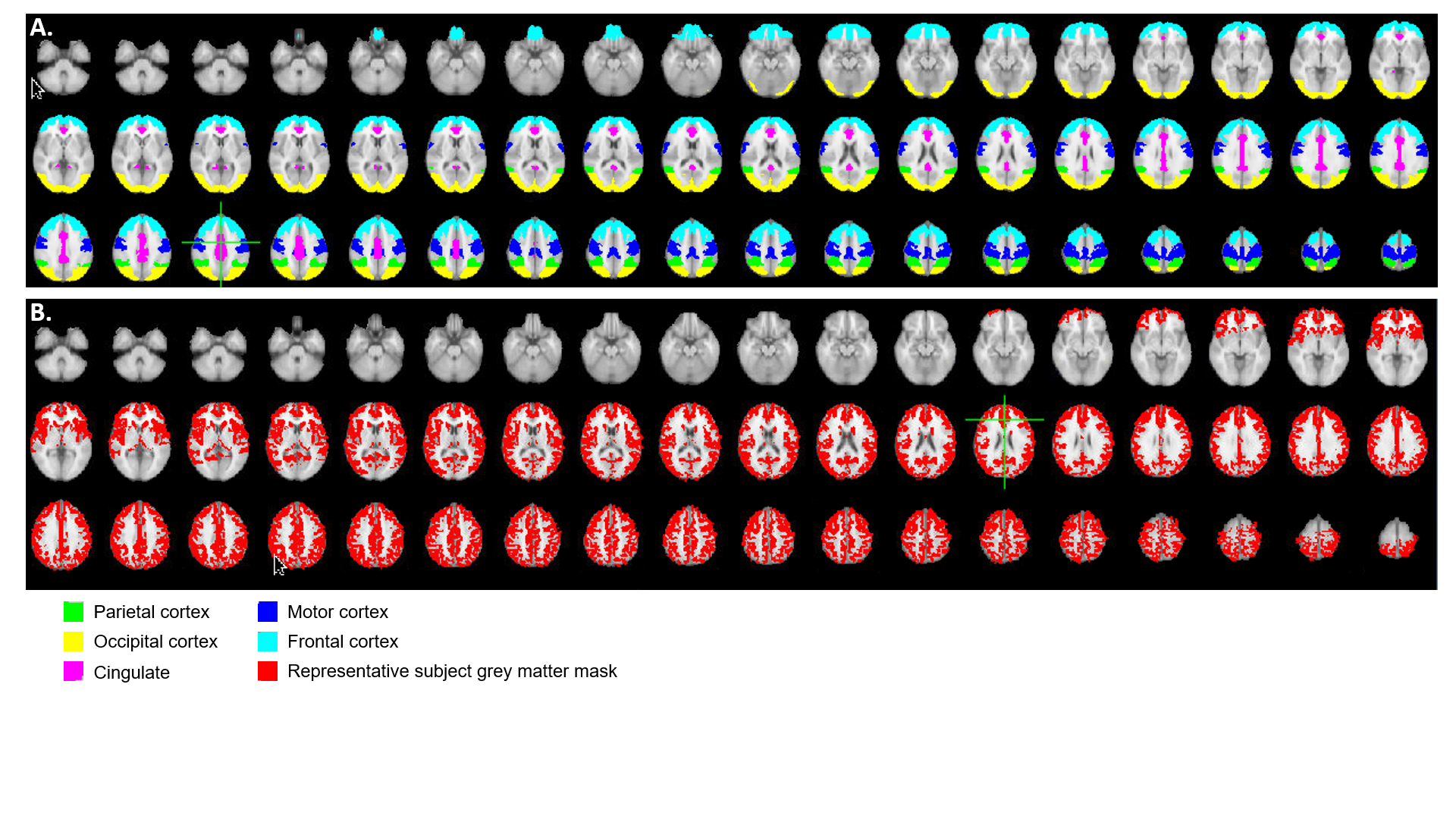


***Figure S1: A.*** *shows the region of interest (RoI) masks;* ***B.*** *shows the grey matter (GM) mask for a representative subject overlaid onto the MNI brain, showing the coverage over which CBF was measured. The CBF metrics were calculated for the conjunction of the GM mask with each RoI mask.*

# **Supplementary Information - Results**

### **SI1: Sub-Set Analysis on Eight Participants Pair-Match for Age and Sex**

Due to the unexpected higher CBF (i.e., TCD-MCAv and ASL-MRI transit times) observed in the younger fit group compared to their unfit counterparts, a separate analysis was performed on a sub-set of the participants who were pair-matched for age and sex (6 males and 2 females). Consistent with the whole group findings (Figure 3 and Table S1 and S2), the younger group still showed that transit times were higher in the fit group compared to the unfit group; specifically showing significance in the whole of the grey matter (*p* = 0.046), with RoI analysis revealing significant effects in the cingulate gyrus (*p* = 0.050), and the occipital lobe (*p* = 0.048). No between group differences were observed in TCD measures for this subset (MCAv: *p* = 0.263 and CVCi: *p* = 0.879).

### **SI2: Regional Variation in MRI Resting CBF Measures**

The effects of aging on MRI resting CBF measures are shown in Figure S1A, including whole grey matter cerebral perfusion and transit times and specified RoIs. The effects of fitness are shown in Figure S1B and Figure S1C for whole grey matter and RoIs, for older and younger groups respectively. Whilst a significant difference in transit time was observed between the younger and older groups over all grey matter, no differences were observed for cerebral perfusion. However, this may be due to differences being region specific. Therefore, region specific effects were investigated in the RoIs.

*Cerebral perfusion in RoIs:* Significant group differences (Figure S1) and correlations (Table S1) were observed between younger and older participants for measures of cerebral perfusion in the occipital lobe (*p* = 0.01). Specifically, perfusion in the occipital lobe was 37% higher in the younger group compared to the older group. The parietal lobe showed a significant group difference of 24% in perfusion between the young and old groups (Figure S1), but a significant linear correlation was not observed (Table S1). Perfusion was also higher in the younger group in the cingulate gyrus (1%), frontal lobe (21%) and motor lobe (10%), though these did not reach significance (Figure S2 left panel). Similar to the whole grey matter cerebral perfusion observations, cerebral perfusion in the RoIs were similar between fitness groups for both younger and older participants (Figure S2B and Figure S2C left panels).

*Transit times for RoIs:* Significant group differences were observed between younger and older participants for measures of transit times in the frontal (*p* = 0.01), motor (*p* < 0.01), and parietal (*p* < 0.01) lobes. Specifically, blood flow was 7%, 9% and 10% faster in the frontal, motor and parietal lobes, respectively, in the younger compared to the older group (Figure S1A right panel), which was mirrored in the other RoIs but did not reach significance. Transit times for the specified RoIs were similar between the fitness groups in the older participants (Figure S1B right), whereas in the younger participants, blood flow was significantly faster in the unfit group than the fit group for all considered RoIs [cingulate gyrus (*p* = 0.01), frontal lobe (*p* = 0.01), motor lobe (*p* = 0.02), occipital lobe (*p* = 0.00) and parietal lobe (*p* = 0.02)] (Figure S2C right). This latter observation was consistent with the MCAv observations for these younger participants (Figure 1).


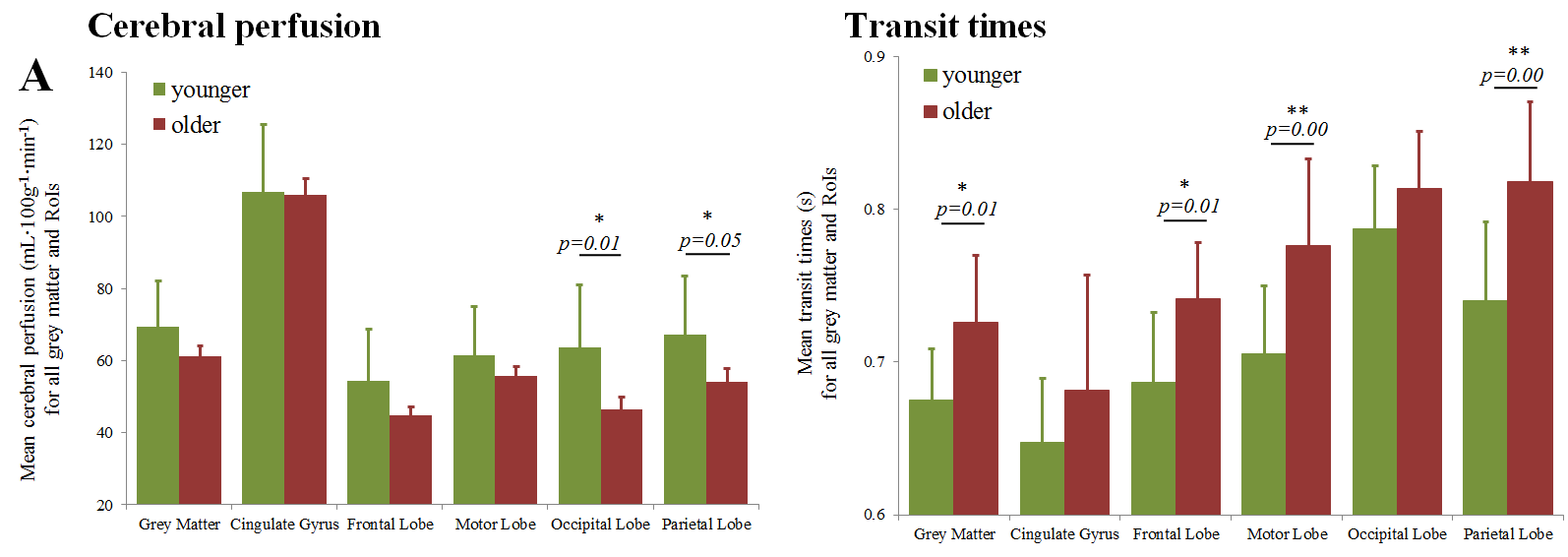

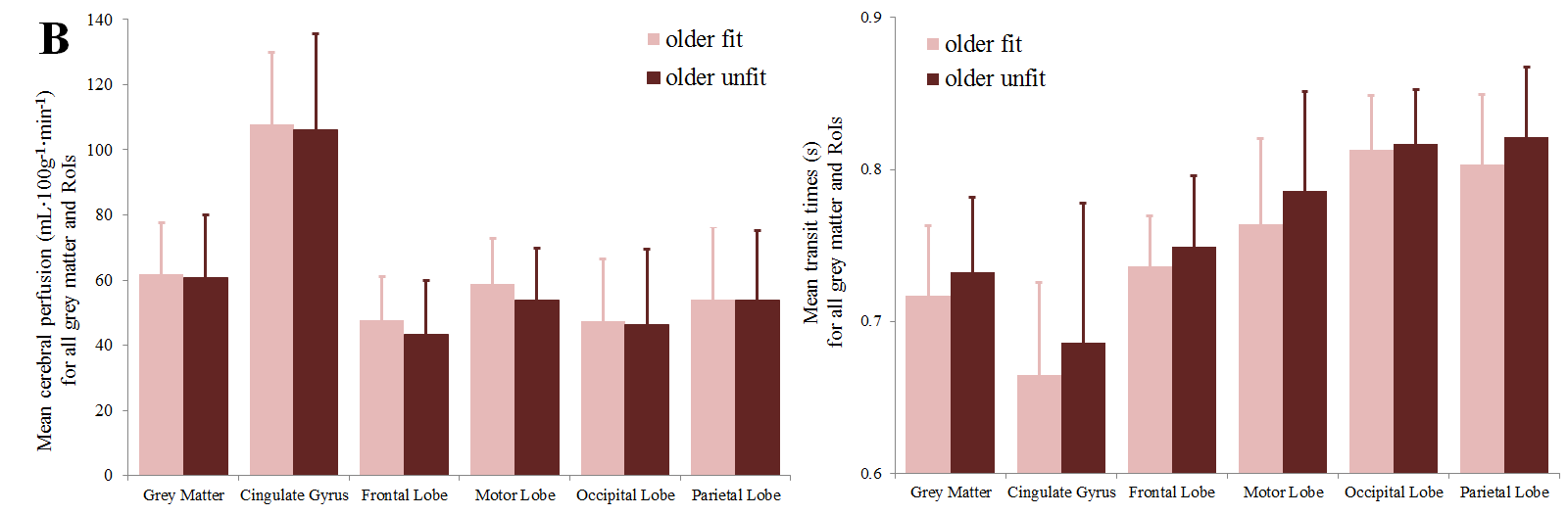


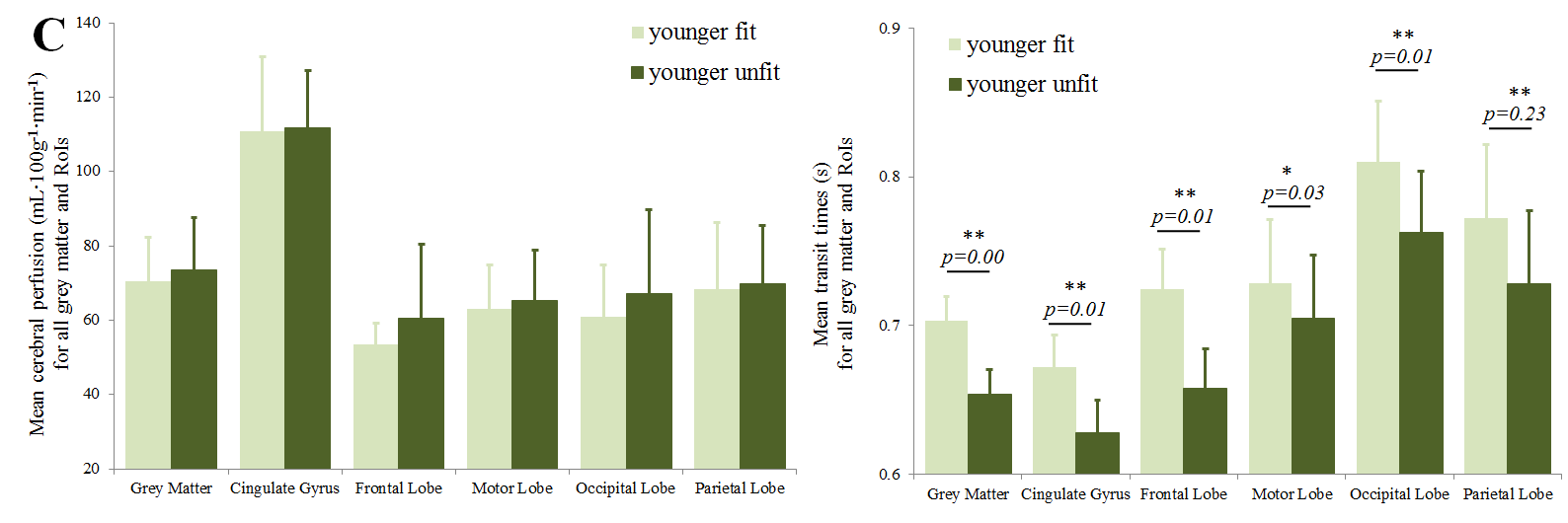
***Figure S2.*** *MRI resting CBF outcome measures of cerebral perfusion (left panel) and transit times (right panel) for all grey matter and specified regions of interest (RoIs). Metrics were separated into:* ***A:*** *Younger and older groups,* ***B:*** *Older fit and unfit groups, and* ***C:*** *Younger fit and unfit groups. Error bars denote standard deviations. Significance values from one-way ANOVA: * p ≤ 0.05; ** p ≤ 0.01. ^t^ Shows a trend towards significance: 0.05 < p ≤ 0.1.*


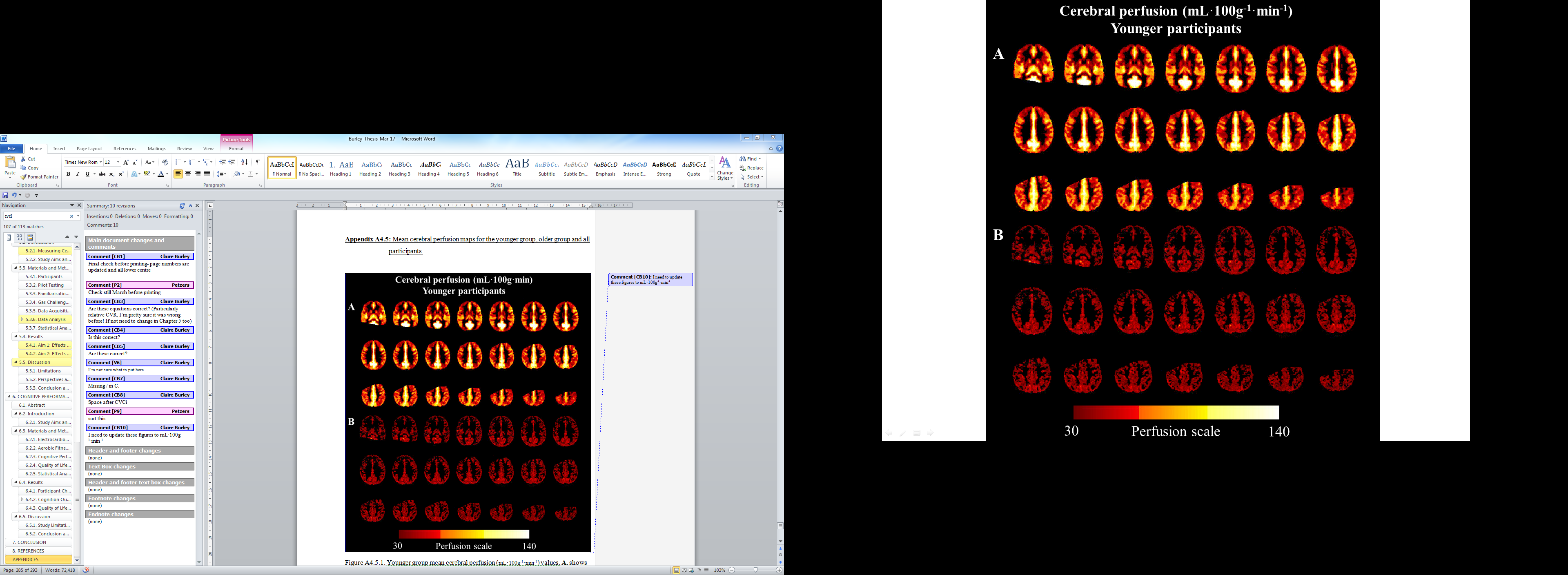


***Figure S3.1.*** *Younger group mean cerebral perfusion (mL·100g^-1^·min^-1^) values.* ***A.*** *shows mean and* ***B.*** *shows standard deviation.*


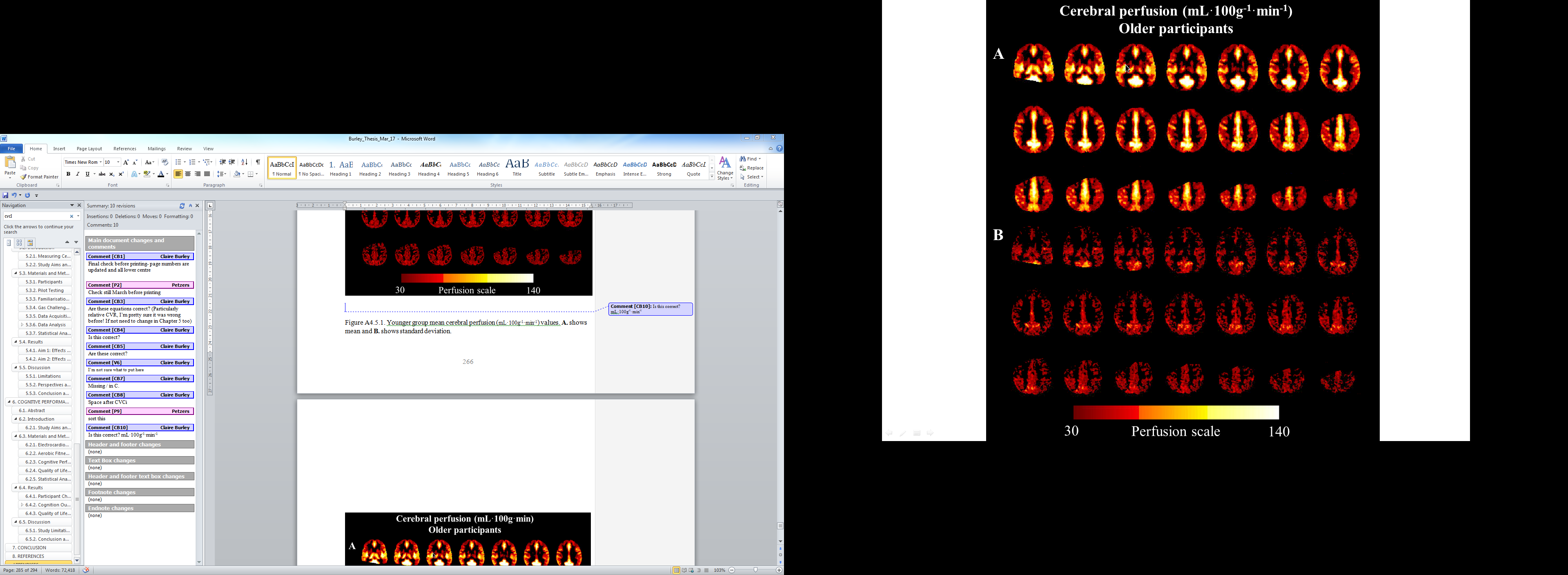


***Figure S3.2.*** *Older group mean cerebral perfusion (mL·100g^-1^·min^-1^) values.* ***A.*** *shows mean and* ***B.*** *shows standard deviation.*


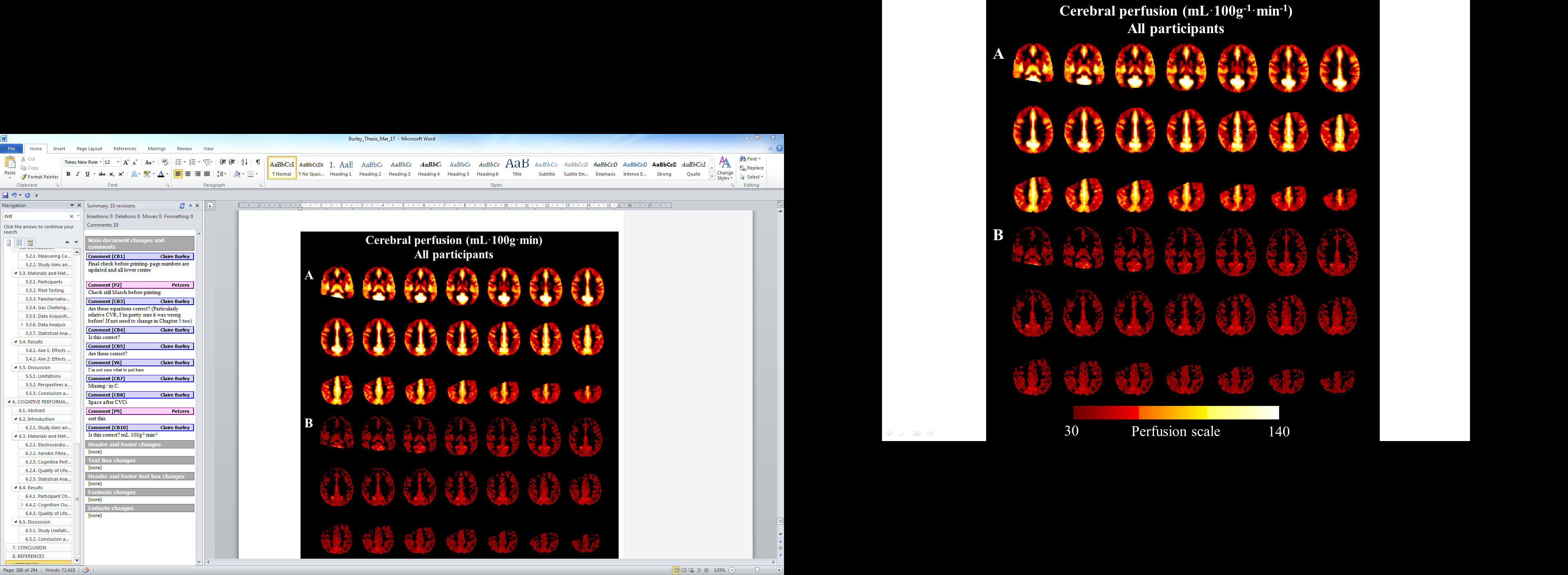


***Figure S3.3.*** *All participants mean cerebral perfusion (mL·100g^-1^·min^-1^) values.* ***A.*** *shows mean and* ***B.*** *shows standard deviation.*

*
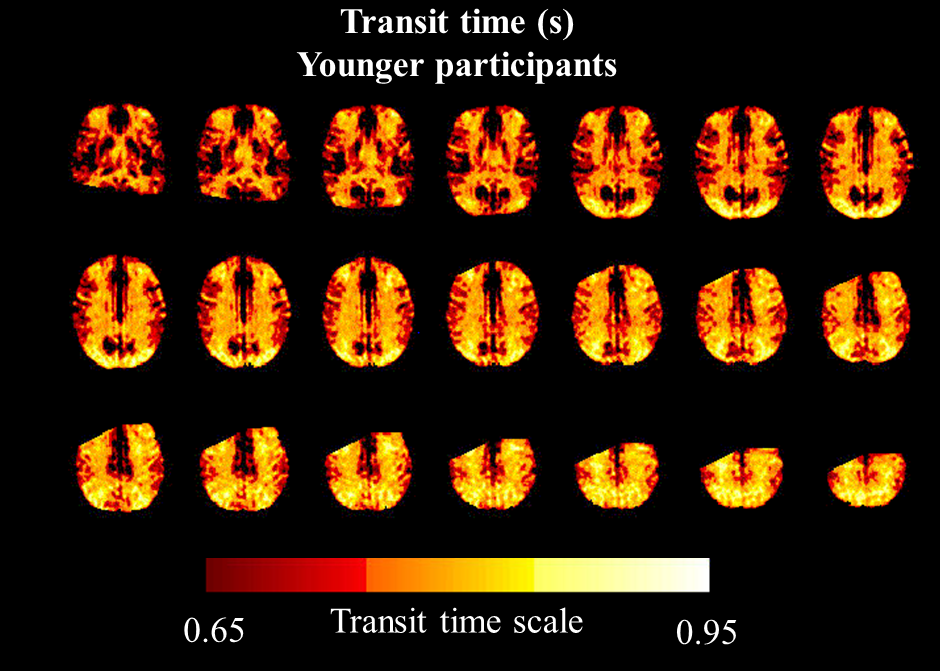
****Figure S4.1.*** *Mean transit time (seconds) maps for all younger participants.*


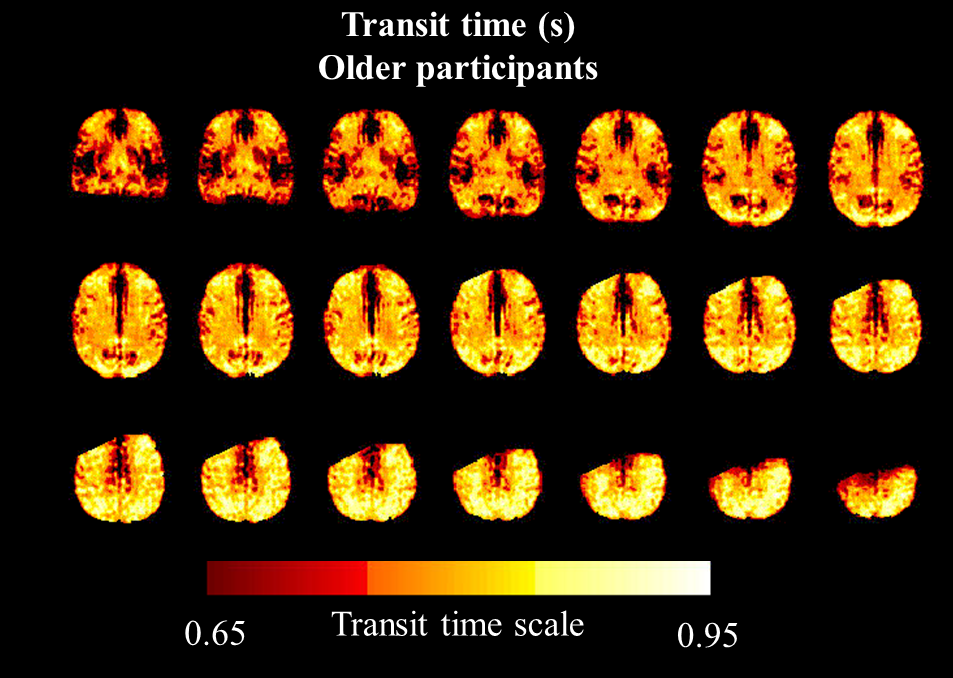


***Figure S4.2.*** *Mean transit time (seconds) maps for all older participants.*


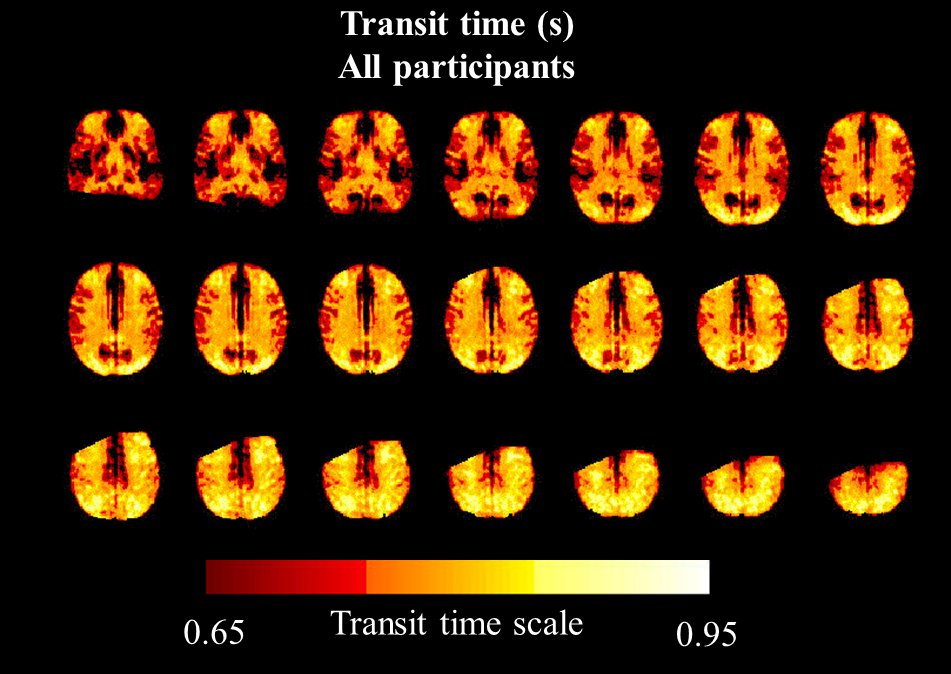


***Figure S4.3.*** *Mean transit time (seconds) maps for all participants.*

### **MRI and TCD CBF Measure Correlations across RoIs**

*Table S1. Correlations of MRI ASL measures of resting CBF across all grey matter and RoIs with age and TCD measures of resting CBF (MCAv and CVCi).*

|  |  |  | **TCD data** |  |
| --- | --- | --- | --- | --- |
|  |  | **Age** | MCAv  (cm·s^-1^) | CVCi  (cm·s^-1^ ·mm Hg^-1^) |
| **Age** |  | _ | **-0.54**** | **-0.59**** |
| **ASL data:** | Grey matter CP (mL·100g^-1^·min^-1^) | -0.23 | 0.12 | 0.21 |
| **Cerebral perfusion** | Cingulate gyrus CP (mL·100g^-1^·min^-1^) | 0.12 | -0.06 | 0.01 |
|  | Frontal CP (mL·100g^-1^·min^-1^) | -0.33 | 0.18 | 0.32 |
|  | Motor CP (mL·100g^-1^·min^-1^) | -0.19 | 0.12 | 0.25 |
|  | Occipital CP (mL·100g^-1^·min^-1^) | **-0.41*** | 0.30 | 0.33 |
|  | Parietal lobe CP (mL·100g^-1^·min^-1^) | -0.33 | 0.19 | 0.25 |
| **ASL data:** | Grey matter TT (s) | **0.61**** | **-0.60**** | **-0.46**** |
| **Transit time** | Cingulate gyrus TT (s) | 0.32 | **-0.35*** | -0.34 |
|  | Frontal TT (s) | **0.64**** | **-0.65**** | **-0.45**** |
|  | Motor TT (s) | **0.62**** | **-0.52**** | **-0.42*** |
|  | Occipital TT (s) | **0.34*** | -**0.29*** | -0.17 |
|  | Parietal TT (s) | **0.63**** | **-0.51**** | -0.32 |

*Values represent Pearson’s r correlations. Significance (2-tailed): * p ≤ 0.05; ** p ≤ 0.01. ^t^ Shows a trend towards significance: 0.05 < p ≤ 0.1. Red numbers show correlations between typical approaches from each modality. Abbreviations: ASL, arterial spin labelling; CP, cerebral perfusion; CVCi, cerebrovascular conductance; MRI, magnetic resonance imaging; MCAv; middle cerebral artery blood velocity; RoI, region of interest; TCD, transcranial Doppler; TT, transit time.*

***Table S2.*** *Correlation (Pearson’s r) of resting CBF measures with age, and fitness (separately for the younger and older group).*

|  | All participants | Younger | Older |
| --- | --- | --- | --- |
|  | Age | Fitness: V̇O2 max | Fitness: V̇O2 max |
| Measure | *r* | *r* | *r* |
| MRI: GM cerebral perfusion (mL·100g^-1^·min^-1^) | -0.231 | 0.099 | 0.071 |
| MRI: GM transit time (s) | **0.606**** | **0.785**** | -0.115 |
| TCD: MCAv (cm·s^-1^) | **-0.535**** | **-0.494*** | **0.520*** |
| TCD: CVCi (cm·s^-1^ ·mm Hg^-1^) | **-0.586**** | -0.250 | **0.732**** |

Values represent Pearson’s *r* correlations. Significant (2-tailed) age/fitness effects: * *p* ≤ 0.05; ** *p* ≤ 0.01. ^t^ Shows a trend towards significance: 0.05 < *p* ≤ 0.1.

*Abbreviations: CBF, cerebral blood flow; MRI, magnetic resonance imaging; TCD, transcranial Doppler; GM, grey matter; MCAv, middle cerebral artery blood velocity; CVCi, cerebrovascular conductance; MAP, mean arterial blood pressure; V̇O2 max, maximum rate of oxygen consumption.*
